# Supplementary material for: In Silico, In Vitro, and In Vivo Investigations on Adapalene as Repurposed Third Generation Retinoid against Multiple Myeloma and Leukemia
Source: Cancers (Basel). 2023 Aug 16;15(16):4136. doi: 10.3390/cancers15164136 (PMC10452460; doi:10.3390/cancers15164136)
Supplement: Supplementary file 1 [file cancers-15-04136-s001.zip › cancers-2492161-supplementary.pdf]

Figure S1.

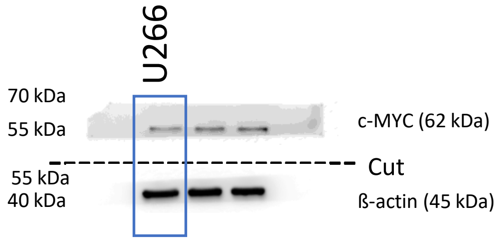

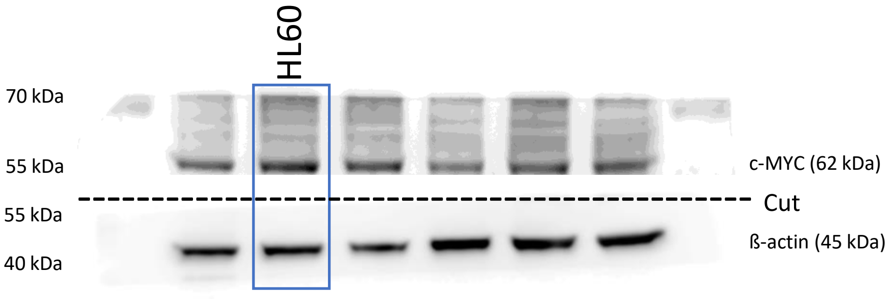

	U266	HL60
c-MYC intensity	73.666	122.993
ß-actin intensity	150.332	79.604
c-MYC/ß-actin	0.490	1.545

Figure S1. Original western blots for Figure 1A. Protein band densitometric analysis was performed by ImageJ software and is shown in the table.

Figure S2.

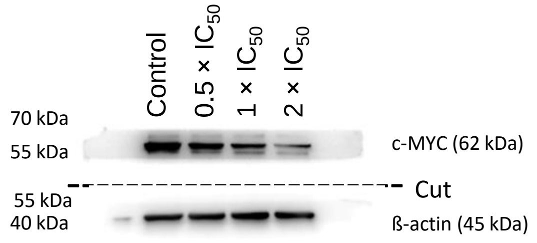

	Control	0.5 × IC_{50}	1× IC_{50}	2 × IC_{50}
c-MYC intensity	104.025	66.549	45.786	26.609
ß-actin intensity	89.794	91.85	116.131	99.676
c-MYC/ß-actin	1.158	0.724	0.394	0.267

Figure S2. Original western blots for Figure 5 A. Protein band densitometric analysis was performed by ImageJ software and is shown in the table.

Figure S3.

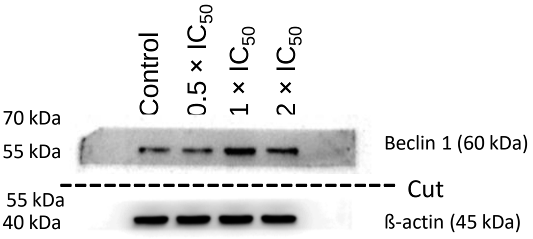

	Control	0.5 × IC_{50}	1× IC_{50}	2 × IC_{50}
Beclin 1 intensity	142.747	145.483	198.498	175.874
ß-actin intensity	182.468	180.702	192.139	184.009
Beclin 1/ß-actin	0.782	0.805	1.033	0.955

Figure S3. Original western blots for Figure 11 C. Protein band densitometric analysis was performed by ImageJ software and is shown in the table.

Figure S4.

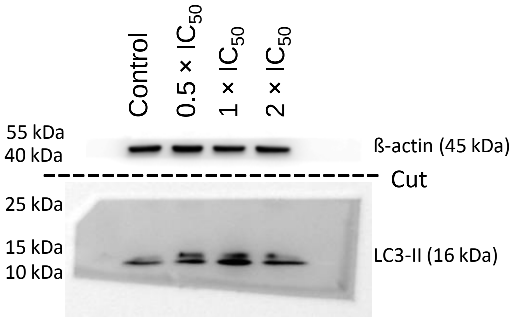

	Control	0.5 × IC_{50}	1× IC_{50}	2 × IC_{50}
LC3-II intensity	133.248	146.029	186.619	150.806
ß-actin intensity	197.102	198.763	189.765	197.078
LC3-II/ß-actin	0.676	0.734	0.983	0.765

Figure S4. Original western blots for Figure 11 C. Protein band densitometric analysis was performed by ImageJ software and is shown in the table.

Figure S5.

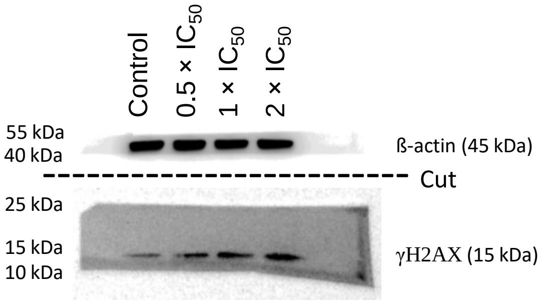

	Control	0.5 × IC_{50}	1× IC_{50}	2 × IC_{50}
γH2AX intensity	92.468	105.603	110.557	134.315
ß-actin intensity	184.332	170.016	159.529	161.216
γH2AX /ß-actin	0.501	0.621	0.693	1.450

Figure S5. Original western blots for Figure 11 C. Protein band densitometric analysis was performed by ImageJ software and is shown in the table.

Supplementary Table S1: Binding affinities (kcal/mol) of the top 170 FDA compounds as identified by PyRx.

Zinc code	Pyr-x binding affinity (kcal/mol)	Zinc code	Pyr-x binding affinity (kcal/mol)	Zinc code	Pyr-x binding affinity (kcal/mol)
ZINC000052955754	-8.03	ZINC000169621200	-7.17	ZINC000004097343	-6.93
ZINC000003978005	-7.90	ZINC000003784182	-7.17	ZINC000070466416	-6.93
ZINC000006716957	-7.73	ZINC000164760756	-7.13	ZINC000169621231	-6.90
ZINC000000537928	-7.70	ZINC000100022637	-7.13	ZINC000169344691	-6.90
ZINC000001612996	-7.70	ZINC000004175630	-7.10	ZINC000014210642	-6.90
ZINC000084668739	-7.60	ZINC000001530886	-7.10	ZINC000148723177	-6.90
ZINC000064033452	-7.60	ZINC000003816287	-7.10	ZINC000169621219	-6.87
ZINC000000896717	-7.57	ZINC000068202099	-7.07	ZINC000030691797	-6.87
ZINC000012503187	-7.53	ZINC000068204830	-7.07	ZINC000001550477	-6.87
ZINC000100073786	-7.50	ZINC000003977978	-7.03	ZINC000005844788	-6.87
ZINC000040430143	-7.40	ZINC000000000903	-7.00	ZINC000003993855	-6.83
ZINC000036701290	-7.37	ZINC000000001370	-7.00	ZINC000004212851	-6.83
ZINC000008101127	-7.33	ZINC000000538312	-7.00	ZINC000016052277	-6.83
ZINC000027428713	-7.33	ZINC000001481956	-7.00	ZINC000100036924	-6.83
ZINC000013831130	-7.27	ZINC000004214700	-7.00	ZINC000034806477	-6.83
ZINC000001996117	-7.27	ZINC000014879992	-7.00	ZINC000053683151	-6.83
ZINC000019796080	-7.23	ZINC000100017856	-7.00	ZINC000169621215	-6.83
ZINC000203757351	-7.23	ZINC000009574770	-6.97	ZINC000019632618	-6.83
ZINC000011679756	-7.23	ZINC000034089131	-6.97	ZINC000000968264	-6.80
ZINC000169621228	-7.20	ZINC000029416466	-6.97	ZINC000000537891	-6.80
ZINC000000601317	-7.20	ZINC000035328014	-6.97	ZINC000012503068	-6.80
ZINC000003925861	-7.20	ZINC000164760874	-6.97	ZINC000066166864	-6.80
ZINC000003939013	-7.17	ZINC000000896557	-6.97	ZINC000096903163	-6.80
ZINC000003938482	-7.17	ZINC000052716421	-6.97	ZINC000003826253	-6.77
ZINC000100014909	-7.17	ZINC000100378061	-6.93	ZINC000004098633	-6.77
ZINC000004099009	-7.17	ZINC000003872566	-6.93	ZINC000003927822	-6.77

Zinc code	Pyr-x binding affinity (kcal/mol)
ZINC000084758235	-6.77
ZINC000095551509	-6.73
ZINC000049783788	-6.73
ZINC000253630390	-6.73
ZINC000169621230	-6.70
ZINC000000538273	-6.70
ZINC000003797541	-6.70
ZINC000004629876	-6.70
ZINC000169621220	-6.70
ZINC000052509366	-6.70
ZINC000003817234	-6.67
ZINC000096006018	-6.67
ZINC000028639340	-6.63
ZINC000000643153	-6.63
ZINC000001493878	-6.63
ZINC000013129998	-6.63
ZINC000043100953	-6.63
ZINC000003921872	-6.60
ZINC000008577218	-6.60
ZINC000014768621	-6.60
ZINC000003977777	-6.60
ZINC000043100709	-6.60
ZINC000094566093	-6.60
ZINC000096006024	-6.60
ZINC000169621223	-6.60
ZINC000011681563	-6.57

Zinc code	Pyr-x binding affinity (kcal/mol)
ZINC000100018598	-6.57
ZINC000003914596	-6.53
ZINC000001529323	-6.50
ZINC000003881958	-6.47
ZINC000118912517	-6.43
ZINC000003824921	-6.37
ZINC000001850377	-6.37
ZINC000000537795	-6.37
ZINC000003813083	-6.33
ZINC000003830716	-6.30
ZINC000011617039	-6.30
ZINC000011616925	-6.17
ZINC000011681534	-6.10

Supplementary table S2: An overview of the molecular docking results of the top 117 FDA compounds as determined by AutoDock 4.2.6 tools. A cutt-off of -6.6 kcal/mol was set and any FDA compound with lower binding energy was identified and its function was determined. Based on their function, we proceeded our study with the compounds highlighted in bold and with red squares.

Zinc code	Lowest binding energy (kcal/mol)	Predicted Ki (μM)	Compound Identity	Application
ZINC000100073786	-8.06	1.23	Ecamsule	Filter out UVA rays
ZINC000001530886	-7.66	2.47	Telmisartan	Management of hypertension
ZINC000084668739	-7.62	2.61	Lifitegrast	Treatment of keratoconjunctivitis sicca
ZINC000003978005	-7.48	3.32	Dihydroergotamine	Treatment of migraines
ZINC000036701290	-7.47	3.50	Ponatinib	Treatment of chronic myeloid leukemia and Philadelphia chromosome–positive acute lymphoblastic leukemia
ZINC000169621219	-7.42	3.66	Eribulin	Anticancer drug
**ZINC000100017856**	**-7.35**	**4.11**	**Mepron**	**Prevention of Pneumocystis pneumonia (PCP)**
ZINC000012503187	-7.33	4.65	Conivaptan	Treatment of hyonatremia
**ZINC000003784182**	**-7.27**	**4.69**	**Differin**	**Treatment of mild-moderate acne**
ZINC000000601317	-7.21	5.27	Difenoxin	Treatment of diarrhea
**ZINC000064033452**	**-7.18**	**5.84**	**Lumacaftor**	**Acts as a chaperone during protein folding and increases the number of CFTR proteins that are trafficked to the cell surface**
ZINC000019632618	-7.17	5.58	Imatinib	Anticancer drug
ZINC000008101127	-7.08	8.75	Indocyanine Green	Medical diagnostics
ZINC000011679756	-6.96	7.99	Eltrombopag	Developed for certain conditions that lead to thrombocytopenia
ZINC000000538312	-6.95	8.04	Risperdal	Antipsychotic medication.
ZINC000003977777	-6.83	9.96	Cyclocort	Treatment a variety of skin conditions
ZINC000003872566	-6.76	12.20	Fexofenadine	Antihistamine pharmaceutical drug
ZINC000003925861	-6.71	12.16	Vorapaxar	Thrombin receptor antagonist
ZINC000052955754	-6.69	12.53	Ergotamine	Treatment of acute migraine attacks
ZINC000003816287	-6.66	13.60	Axitinib	Small molecule tyrosine kinase inhibitor
ZINC000006716957	-6.61	14.45	Nilotinib	Anticancer drug

ZINC000030691797	-6.60	14.52	Perampanel	Antiepileptic drug

Zinc code	Lowest binding energy (kcal/mol)	Predicted Ki (μM)	Zinc code	Lowest binding energy (kcal/mol)	Predicted Ki (μM)
ZINC000164760756	-6.58	15.17	ZINC000011617039	-5.96	43.36
ZINC000001493878	-6.54	16.82	ZINC000000538273	-5.94	44.00
ZINC000164760874	-6.54	16.63	ZINC000014768621	-5.94	45.62
ZINC000066166864	-6.53	16.24	ZINC000003993855	-5.92	46.00
ZINC000100014909	-6.53	17.70	ZINC000052716421	-5.90	47.48
ZINC000000968264	-6.51	17.03	ZINC000003797541	-5.85	51.50
ZINC000003977978	-6.38	21.09	ZINC000013129998	-5.84	52.60
ZINC000043100709	-6.35	25.46	ZINC000001481956	-5.83	54.35
ZINC000070466416	-6.32	25.69	ZINC000000537891	-5.81	55.01
ZINC000001612996	-6.34	22.50	ZINC000052509366	-5.78	57.80
ZINC000148723177	-6.27	25.23	ZINC000096903163	-5.77	58.57
ZINC000034089131	-6.26	25.57	ZINC000019796080	-5.73	67.12
ZINC000203757351	-6.26	27.67	ZINC000012503068	-5.72	64.26
ZINC000068202099	-6.19	30.20	ZINC000004175630	-5.72	66.60
ZINC000004214700	-6.18	29.49	ZINC000000537928	-5.71	67.80
ZINC000043100953	-6.16	35.92	ZINC000169621215	-5.64	73.57
ZINC000040430143	-6.11	35.04	ZINC000001996117	-5.61	78.33
ZINC000035328014	-6.11	33.21	ZINC000014879992	-5.56	84.62
ZINC000003927822	-6.08	40.42	ZINC000011681563	-5.54	86.64
ZINC000000000903	-6.04	37.32	ZINC000003817234	-5.53	90.59
ZINC000000001370	-6.04	37.45	ZINC000034806477	-5.51	91.49
ZINC000003826253	-6.03	37.82	ZINC000100018598	-5.48	95.60
ZINC000169344691	-6.02	39.01	ZINC000004629876	-5.45	101.44
ZINC000003881958	-5.98	41.11	ZINC000118912517	-5.44	102.85
